# Identification of railway subgrade defects based on ground penetrating radar

**DOI:** 10.1038/s41598-023-33278-w

**Published:** 2023-04-13

**Authors:** Zhezhe Hou, Weigang Zhao, Yong Yang

**Affiliations:** 1 School of Material Science and Engineering, Shijiazhuang Tiedao University, Shijiazhuang, China; 2 School of Safety Engineering and Emergency Management, Shijiazhuang Tiedao University, Shijiazhuang, China

**Keywords:** Civil engineering, Geophysics

## Abstract

A recognition method is proposed to solve the problems in subgrade detection with ground penetrating radar, such as massive data, time–frequency and difference in experience. According to the sparsity of subgrade defects in radar images, the sparse representation of railway subgrade defects is studied from the aspects of the time domain, and time–frequency domain with compressive sensing theory. The features of the radar signal are extracted by sparse representation, thus the sampling data are reduced. Based on fuzzy C-means and generalized regression neural network, a rapid recognition of the railway subgrade defects is realized. Experimental results show that the redundancy of data is reduced, and the accuracy of identification is greatly increased.

## Introduction

Railway subgrades have long been influenced by the environment, climate conditions, and trainloads. Some subgrade defects are inevitable, such as sinkholes, settlements, mud pumping, and affect traffic safety and effective maintenance. Therefore, railway subgrade defects should be accurately and effectively detected to ensure the railway subgrade work properly.

With rapidity, continuity, and high accuracy, GPR technology satisfies the requirements for the detection of the continuous detection of railway subgrades^1–3^. Liu et al.^4^ utilized the different GPR antennas and frequencies to detect ballast layer. Tosti et al.^5^ used GPR systems equipped with central frequencies of 600, 1000, 1600 and 2000 MHz to obtain the dielectric permittivity of the ballast system. Bi et al.^6^ fused multi-frequency data into a synthetic data to obtain both high resolution and deep penetrating ability. Therefore, multi-frequency antennas have been used more recently. The detections in railway subgrade have been developed^7,8^. Kuo^9^ investigated mud pumping distributions, Huang^10^ analyzed void signals based on the digital images and proposed a void recognition algorithm for subgrade defects. Barrett et al.^11^ considered the degrees of ballast fouling and moisture content, and proposed the measurement of ballast fouling conditions. However, the type, size, and position of subgrade defects are different. Consequently, the GPR echo signals are affected, and identification becomes complex^12,13^.

Feature extraction and target recognition are the key points in the research of GPR nondestructive testing^14^. The identification of subgrade defects based on GPR has been concerned for decades^15,16^. Generally, the research is mainly the echo signal and GPR image. Fabio et al.^17^ identified the underground targets with the shape and position of the echo signal. Ciampoli et al.^18^ analyzed the relationship between the aggregates grain size and the frequency spectra peaks, and assessed the railway ballast geometric properties. Therefore, this method works efficiently in a certain geometry (mainly shape) or single layer, but in subgrade defects, nonhomogeneous defects change the shape of the echoes and affect detection accuracy. Li et al.^19^ used finite difference time domain to classify hard objects. Fontul et al.^20^ considered the relationship between the electromagnetic properties and the ballast water content, applied the frequency domain analysis to assess the ballast condition. Liu et al.^21^ processed the GPR signals in the time and frequency domains, and effectively accessed ballast fouling and moisture content. Ciampoli et al.^22^ estimated electromagnetic parameters of railway ballast. Zhang et al.^23^ utilized time–frequency features of GPR signal to evaluate the pavement conditions. And wavelet transform (WT) is provided for the echo feature extraction. Sadeghi et al.^24^ applied WT was to interpret GPR data to evaluate ballast fouling. Ciampoli et al.^3^ used both time–frequency and discrete wavelet techniques to evaluate the levels of fouled ballast. The methods are limited to process a large number of original data and obtain redundant feature parameters. Therefore, time lags are a considerable drawback in these methods, which are contradictory to rapid evaluation the subgrade condition.

With the development of feature extraction methods, such as compressed sensing (CS), sparse representation, a new method is provided to extract features. Shao et al.^25,26^ analyzed the relationship between the frequency and standard deviation, obtained the sparse feature vector of ballasted railways. Sun et al.^27^ combined sparse scattering with geometrical features of landmines, detected the landmine rapidly. Based on previous studies, this method is a clear advantage in sparsity for the ballast layer or single structure, and obviously reduces the amount of data. Our study focuses on identifying the complex heterogeneous subgrade defects, analyzes the features of target echoes, and constructs a feature extraction to identify subgrade defects. "Methodology" Section analyzes the sparse characteristics of the spatial structure, introduces a methodology to identify subgrade defects. "Result analysis and discussion" Section describes a rapid identification of railway subgrade defects based on GPR images, and verifies the reliability of the proposed algorithm through field experiments. "Conclusion" Section summarizes our study and presents relevant conclusions.

## Methodology

### Target sparsity and sparse imaging

For radar signals of fixed frequency, the mixer output is a linear frequency modulation signal, and its frequency signal is as follows:

1 $$ S_{T} \left( t \right) = Ae^{{ - j2\pi \left( {f_{0} t + \frac{1}{2}kt^{2} } \right)}} $$

where *A* is the amplitude of the signals, $f_{0}$ is the initial frequency, and $k$ is the frequency modulation slope. The echo of a point with a distance of *H* is as follows:

2 $$ S_{H} \left( t \right) = A\sigma e^{{ - j2\pi \left( {f_{0} t + \frac{1}{2}kt^{2} } \right)\left( {t - 2H\left( {\rho ,i} \right)/c} \right)}} /S\left( {H\left( {\rho ,i} \right)} \right) $$

where $\rho$ is the target location, $S_{\left( H \right)}$ is the attenuation factor,$c$ is the electromagnetic wave velocity in a vacuum,$i$ is the echo channel number, and $\sigma$ is the target reflection coefficient.

To speed up signal reading and processing speed, every 25 channels of radar signals form a 256 × 256 pixel image $\phi \left[ {\mu_{x} ,\mu_{y} } \right]^{{}}$.The coincidence rate of radar images is 50%. Combined with the sparsity of railway subgrade defects, the relationship between the measurement target and space images is as follows:

3 $$ d\left( {\mu_{x} ,\mu_{y} ,f} \right) = \psi \pi_{T} \left( {x,y,z} \right) $$

where $\pi_{T} \left( {x,y,z} \right)$ is the spatial position of the measurement target, $d\left( {\mu_{x} ,\mu_{y} ,f} \right)$ is the frequency-space image, and $\psi$ is the space transformation basis matrix, that is, the dictionary.

### Sparse matrix of radar signals

To establish the sparse matrix, the measurement target must be discrete in spatial position,*i* is the echo channel number, $\left( {x_{i} ,y_{i} ,z_{i} } \right)$ is the spatial position, image space $B$ is formed correspondingly by* N* pixels $\left\{ {\pi_{1} ,\pi_{2} , \ldots ,\pi_{n} } \right\}$, and each pixel $\pi_{i}$ corresponds to the three-dimensional vector $\left( {x_{i} ,y_{i} ,z_{i} } \right)$. The ith vector radix of pixel $\pi_{i}$ is as follows:

4 $$ \left[ {\psi_{i} } \right]_{j} = \exp \left[ { - j\omega \left( {t - \Gamma_{i} \left( {\pi_{j} } \right)} \right)} \right]^{{}} $$

where $\omega$ is the frequency vector *B*, and the dictionary matrix corresponding to the echo channel is obtained through Formula (4). The $P$ target echo is received by the ith echo channel

5 $$ \zeta_{i} \left( \omega \right) = \sum\nolimits_{k = 1}^{P} {b\left( k \right)} \exp \left[ { - j\omega \left( {t - \Gamma_{i} \left( \pi \right)_{k} } \right)} \right] $$

Formula (6) is converted to a vector

6 $$ \varsigma_{i} \left( \omega \right) = \psi_{i} b $$

where $b$ is the weighted steering vector of the target space, $\pi_{j}$ is the partial position of the measurement target, $b_{j} = {{A\sigma_{j} } \mathord{\left/ {\vphantom {{A\sigma_{j} } {\Gamma \left( {\pi_{j} } \right)}}} \right. \kern-0pt} {\Gamma \left( {\pi_{j} } \right)}}$; otherwise, $b_{j} = 0$.

Assume that the collected radar signals are made up of huge amounts of one-dimensional signals of length *L* with a sparsity of* k* (that is, it contains *k* nonzero values), which form a large quantity of sample data. Due to the sparsity of railway subgrade defects in space, this paper proposes the compressive sensing method for data sampling, that is, a small number of signals represent all signals, to construct the target images. The measurement matrix should be a random matrix that is not related to the dictionary. In this paper, the Bernhard matrix composed of 0 and 1 elements is selected as the observation matrix. Therefore, *M* random rows are extracted from the $L \times L$ identity matrix to obtain the measurement matrix $\varphi_{i}$ corresponding to the ith echo channel. The measured value is

7 $$ \beta_{i} = \varphi_{i} \zeta_{i} = \varphi_{i} \psi_{i} b $$

The measurement matrix $\varphi_{i}$ of each echo channel is different.

To obtain the space guidance vector *b*, echo channels *K* are selected. The dictionary matrix is $\psi = \left[ {\psi_{1}^{T} ,\psi_{2}^{T} \ldots ,\psi_{K}^{T} } \right]$, the measurement matrix is $\varphi = diag\left[ {\varphi ,\varphi_{2} \ldots ,\varphi_{K} } \right]$, the measurement value is $\beta = \left[ {\beta_{1}^{T} ,\beta_{2}^{T} \cdot \cdot \cdot ,\beta_{K}^{T} } \right]$, and reconstruction *b* becomes a constrained problem to solve the convex optimization problem.

8 $$ {\hat{\text{b}}} = {\text{argmin}}\left\| {\text{b}} \right\|_{1} \;{\text{s}}.{\text{t}}.\;\beta = \varphi \psi b $$

where Formula (8) is only in the absence of noise. The measured value corresponding to the ith echo channel with noise:

9 $$ \beta_{i} = \varphi_{i} \xi_{i} = \varphi_{i} \psi_{i} b + \mu_{i} $$

where $\mu_{i} = \varphi_{i} n_{i} \sim N\left( {0,\sigma^{2} } \right)$ and $n_{i} \sim N\left( {0,\sigma^{2} } \right)$ is aliasing noise. Convex optimization of the improved $L1$ norm under constraint conditions:

10 $$ \hat{b} = \arg \min \left\| b \right\|_{1} $$

where $A = \varphi \psi$, $\varepsilon = \sigma \sqrt {2\lg N}$. Formula (10) can be used to create the target images.

### Feature representation of railway subgrade defects

The subgrade defects are sparse from the graphical distribution based on the CS (compressed sensing) algorithm. The identification parameters of typical defects are as follows:

1. Peaks of the multiscale wavelet energy spectrum of the subgrade;
2. The time-domain features, such as energy per block, the variation per block, the variation and the demixing points per block.

#### Feature extraction of GPR signals based on the time domain

Based on the continuity and disorder of the phase axes of the subgrade, the time domain characteristics of the subgrade defects are established. A signal of length N is divided into M blocks, and each block image is divided into K segments by length. The coincidence rate between the images is 50%. The features of subgrade are as followed:

11 $$ E_{i} = \sum\limits_{{j = M \cdot {i \mathord{\left/ {\vphantom {i {2 + 1}}} \right. \kern-0pt} {2 + 1}}}}^{{M \cdot {i \mathord{\left/ {\vphantom {i {2 + M}}} \right. \kern-0pt} {2 + M}}}} {A_{j}^{2} } $$

12 $$ \sigma_{i}^{2} = \frac{1}{M - 1}\sum\limits_{j = M \cdot i/2 + M}^{M \cdot i/2 + M} {\left( {A_{j} - \overline{{A_{i} }} } \right)} $$

where $i = 0,1,2, \cdots K - 1$; $E_{i}$ is the energy of the ith segment; $A_{j}$ is the amplitude of the jth sample; $\sigma^{2}$ is the sample variance of the ith segment; $\overline{{A_{i} }}$ is the mean amplitude of the ith segment.

#### Horizontal energy spectrum

The characteristics of the subgrade radar signal are different at each scale. Each scale energy has different contributions to the total energy. The main part of the signal is identified according to the characteristics of the energy spectrum. The component energy of wavelet decomposition at the Jth scale is shown as follows:

13 $$ E_{J}^{A} f\left( n \right) = \sum\limits_{n = 1}^{N} {\left( {A_{J} f\left( n \right)} \right)^{2} } $$

14 $$ E_{J}^{D} f\left( n \right) = \sum\limits_{n = 1}^{N} {\left( {D_{J} f\left( n \right)} \right)^{2} } \;j = 1,2, \ldots ,J $$

where *A*_*J*_* f*(*n*) is the low-frequency reconstructed signal at the *Jth* wavelet decomposition, and *D*_*J*_* f*(*n*) is the high-frequency reconstructed signal at the *Jth* wavelet decomposition.$E_{J}^{A} f\left( n \right)$ and $E_{J}^{D} f\left( n \right)$ are the low- and high-frequency signal energies, respectively, at the *Jth* wavelet decomposition.

#### Sparse matrix

**Figure 1:**
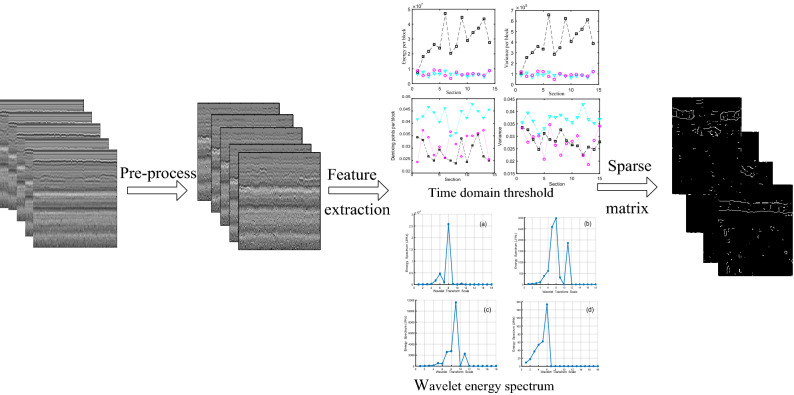
The flow of subgrade defects.

Based on L1-norm optimization method, the training samples matrix are constructed by eigenvalues of subgrade defects and all used as the data dictionary of the sparse representation. The flow of the subgrade defects is shown in Fig. 1.

### Target detection and identification method

#### Identification of subgrade defects based on FCM

The FCM algorithm was as follows:

1. The subgrade defects are divided into three categories: sinkhole, mud pumping, and settlement, and the fuzzy weight index is determined;
2. The clustering center (*v*) and individual fuzzy membership matrix (*u*) of each kind of subgrade defect are set; thus, fuzzy clustering is analyzed;
3. The subgrade defects are classified based on the clustering, the corresponding mean value center (*v*) is obtained, and the distance between the individual (subgrade defect type) in the class and the mean value center is obtained; thus, the fuzzy clustering statistical results are obtained accordingly.

#### Identification of subgrade defects based on FCM-GRNN

**Figure 2:**
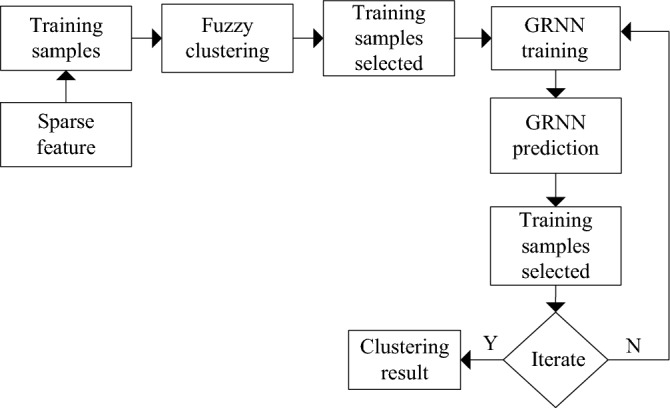
The FCM-GRNN algorithm.

According to the fuzzy boundaries and considerable data of railway subgrade defects, FCM and GRNN algorithms are combined to identify the subgrade defects, as shown in Fig. 2. The specific algorithm is as follows:

1. Based on the FCM, the GRNN is used to predict the type of training samples;
2. The corresponding mean value center (*v*) and the distance between the individual (subgrade defect type) in the class and the mean value center are recalculated, and the data closest to the center are selected as the training samples of the network;
3. After repeated calculations, the final network cluster is obtained.

## Result analysis and discussion

**Figure 3:**
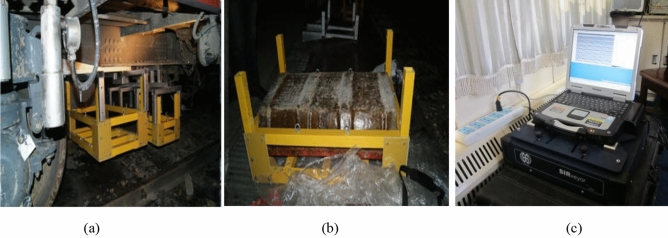
The GPR and its suspension system. (**a**) suspension system of radar antenna for track inspection train, (**b**) photo for fixed radar antenna, (**c**) SIR-20 ground penetrating radar system.

Some sections of the Daqin and Shichang railways are detected by GPR installed at the bottom of rail inspection vehicle, as shown in Fig. 3a. To meet the requirements of maximum detection depth and depth resolution, 100 and 400 MHz radar antennas are adopted to detect the railway subgrade, as shown in Fig. 3b. The GPR system (Fig. 3c) and the working parameters of the ground-penetrating radar are set as follows: sampling interval is 5 cm; the maximum depth reaches 8 m; depth resolution is up to 0.2 m; sampling rate is set to 100 scans^−1^, and a large quantity of railway subgrade defects are sparse in radar images. Therefore, railway subgrade defects meets the requirements of sparse theory.

### Feature extraction of GPR signals

Based on the GPR signal data, the feature extraction method consists of the following two steps: (1) time domain, (2) horizontal energy spectrum. The feature extraction methods of the signals are explained through an example in the following paragraphs.

1. Time domain

Based on the continuity and disorder of the phase axes of the subgrade, the time domain characteristics of the subgrade defects are established.

**Figure 4:**
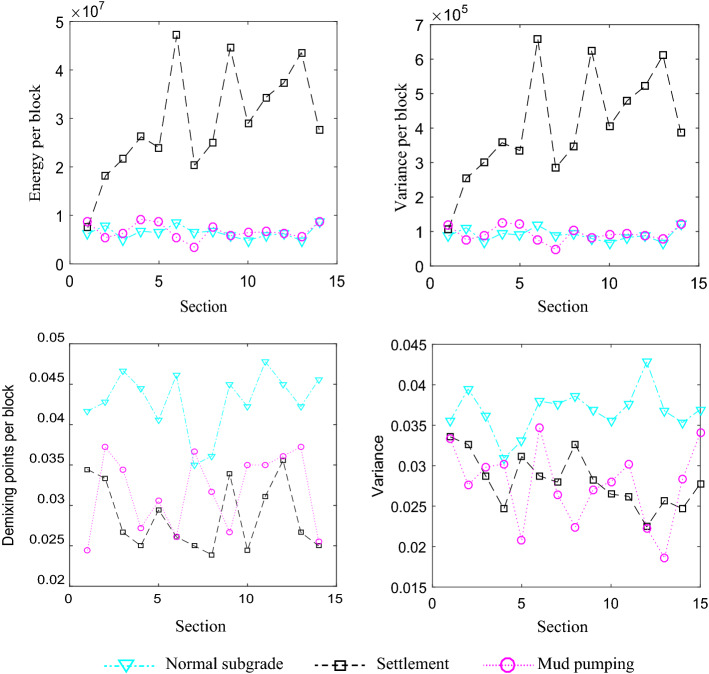
Eigenvalue curve.

The change of the energy and the phase axis of is obvious in the subgrade defects, and the space location, energy, and variation of defects are different from those of the normal subgrade, as shown in Fig. 4. The energy per block and the variation per block can distinguish the normal subgrade from the defects. The phase axes of settlement apparently decline, the energy of the fault increases obviously, and the variation and demixing points per block are distinguished from the settlement. However, the interfaces of mud pumping become vague. In addition, the high conductivity of the mud pumping makes the energy of the radar image low. Judging from the energy and variance of the radar images, the subgrade defects can be identified.

(2) Horizontal energy spectrum

**Figure 5:**
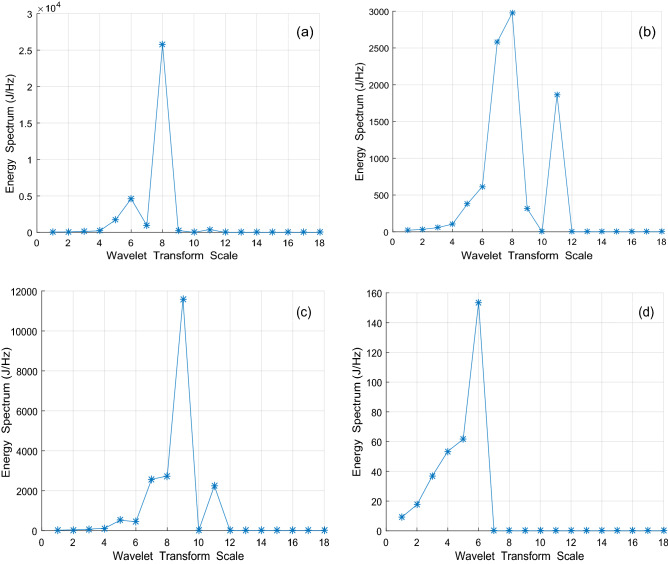
Multiscale wavelet energy spectrum of subgrade. (**a**) the normal subgrade; (**b**) subgrade settlement; (**c**) subgrade sinkhole; (**d**) mud pumping.

The wavelet energy spectrum in scale 18 was built to reduce sample data combining wavelet multi-scale decomposition and power spectra analysis, as shown in Fig. 5. Through the energy spectrum, it can be seen that the characteristic peaks of the normal subgrade, sinkhole, and settlements are all in scale 8, and the characteristic peak of mud pumping is in scale 6. The energy spectrum of the normal subgrade is as high as 2.5 × 10^–4^ J/Hz, and the energy spectrum of mud pumping is as low as 160 J/Hz. The energy spectra between settlement and sinkholes are so similar that it is difficult to distinguish between them.

(3) Analysis of sparsity

**Figure 6:**
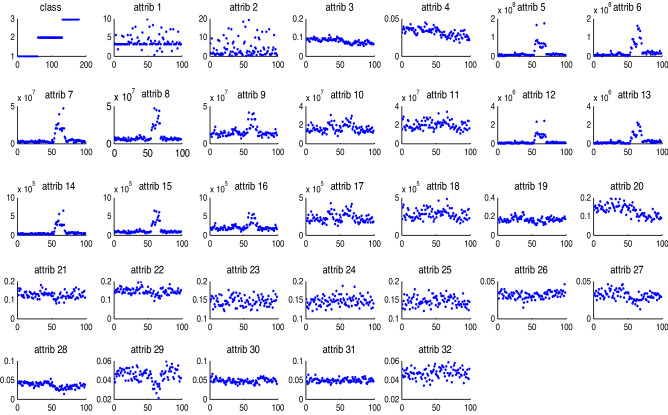
Different dimensional visualization of subgrade defects.

100 blocks of subgrade defects are selected as the test samples, and grouped into three categories: sinkhole, mud pumping, and settlement, corresponding to the first, second and third category. The number of features is 73 extracted by time domain and energy spectrum. The different dimensional visualization of subgrade defects is shown in Fig. 6. All the extracted 32-dimensional eigenvalues are clustered, and used as feature vectors.

**Figure 7:**
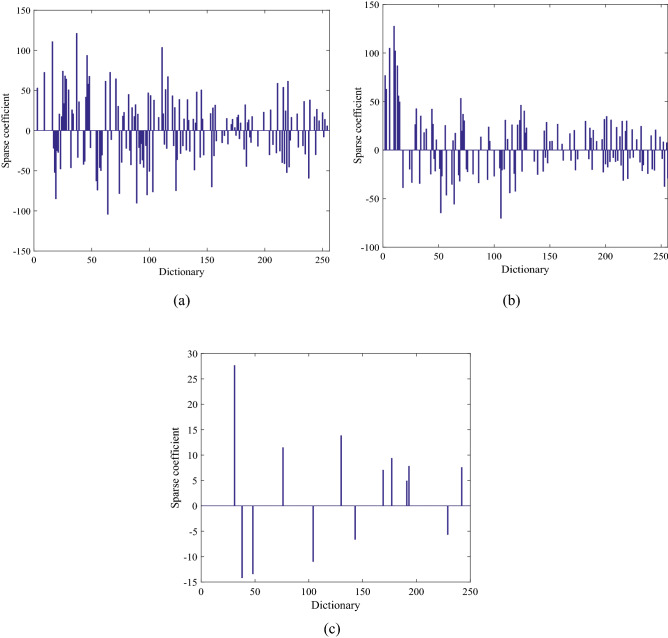
Sparse representation coefficient of settlement in subgrade defects dictionary. (**a**) Sparse coefficient of settlement in dictionaryA^2^, (**b**) Sparse coefficient of settlement in dictionaryA^3^, (**c**) Sparse coefficient of settlement in dictionaryA^1^.

Take subgrade settlement for example, the sparsity in subgrade defects dictionary is analyzed, as shown in Fig. 7. Based on L1 minimum norm method, the sparse coefficient of settlement is calculated in dictionary settlement matrixA^1^, sinkhole matrixA^2^, and mud pumping matrix A^3^, respectively, and thus the dictionary matrix A is made up of the 32-dimensional eigenvalues. The settlement in dictionary A^1^ is sparse, and most of coefficient is 0, as shown in Fig. 7c, but it is not sparse in dictionary A^2^ or A^3^, as shown in Fig. 7a and b.

**Figure 8:**
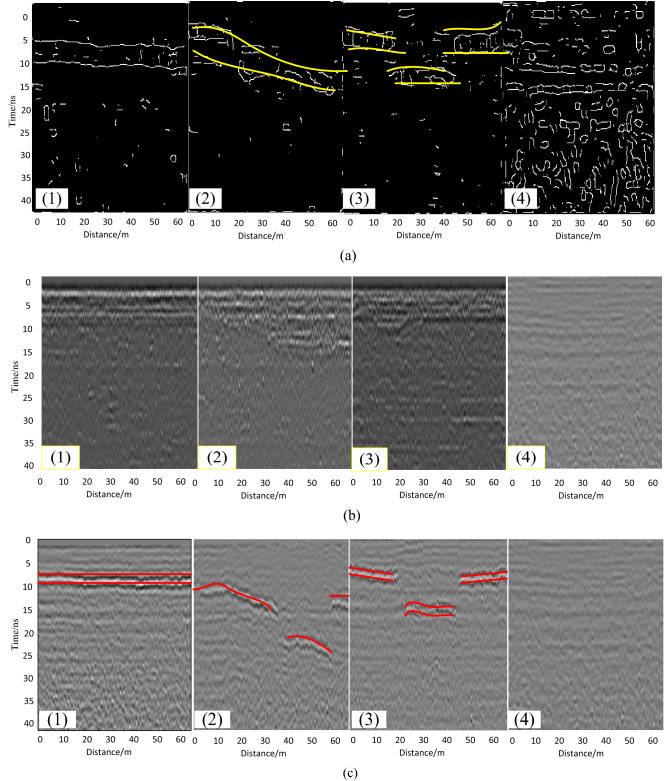
The radar images of subgrade: (**a**) CS images, (**b**) restored images, (**c**) original radar images, including (1) normal subgrade, (2) settlement, (3) sinkhole, (4) mud pumping, respectively.

Compared with CS images, restored images, and original radar images, the feasibility and accuracy of sparse representation is shown in Fig. 8. The radar images of subgrade, including normal subgrade, settlement, sinkhole, mud pumping, are shown in Fig. 8a. The data sets obviously are declined. Restored images based on sparse representation are shown in Fig. 8b, and partial data loss has little effect on CS imaging results. Compared with Fig. 8b and c, it is not difficult to find that the CS algorithm completes the target detection.

### Identification of subgrade defects

**Figure 9:**
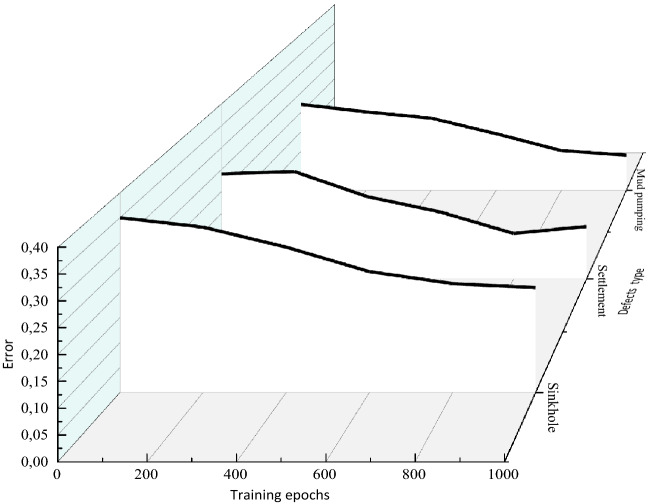
Effect of training epoch on FCM-GRNN test performance.

**Figure 10:**
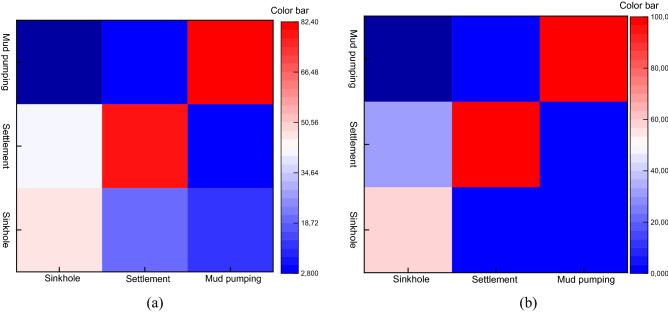
Confusion matrices based on the testing dataset for FCM and FCM-GRNN algorithm, respectively. The X-axis labels are the ground truth labels and the Y-axis labels are the predicted labels. (**a**) FCM algorithm, (**b**) FCM-GRNN algorithm.

Figure 9 shows that the faster convergency with FCM-GRNN algorithm on the basis of the clustering center (*v*) and individual fuzzy membership matrix (*u*) trained by FCM algorithm**.** The training error is converged gradually. Figure 10 shows the classification accuracy obtained by FCM and FCM-GRNN, respectively. Detailed information about the recognition rates is shown in the confusion matrices. The confusion matrices demonstrate that the recognition rates vary significantly (Fig. 10a), and the recognition rates vary weakly (Fig. 10b), thereby, the classification results are influenced by recognition methods. Thus, we can conclude that the FCM-GRNN exhibits higher classification accuracy, and efficient classification of subgrade defects is not implemented by FCM. The clustering center (*v*) and individual fuzzy membership matrix (*u*) are obtained by FCM, then *v* is recalculated and the new *u* is obtained by FCM-GRNN, thus clustering results are improved.

The Daqin Railway subgrade is chosen as the target. A total of 1084 subgrade sinkholes, 970 mud pumping defects, and 1534 subgrade settlements are selected as the test samples. Table 1 lists that railway subgrade defects are effectively identified by the FCM and FCM-GRNN algorithms. The accuracy rate of FCM-GRNN algorithms reaches 100% both for settlement and mud pumping. The accuracy rate of subgrade sinkholes by FCM-GRNN is 59.1%, and the result of it is more accuracy than the result that gain by FCM.

**Table 1:** The accuracy recognition of railway subgrade defects.

Input	Number/channels	Output	Number/channels		Classification accuracy	
			FCM	FCM-GRNN	FCM (%)	FCM-GRNN (%)
Sinkhole	1084	Sinkhole	493	641	45.5	59.1
		Settlement	443	345		
		Mud Pumping	148	98		
Mud pumping	970	Sinkhole	128	0	82.3	100
		Settlement	44	0		
		Mud Pumping	798	970		
Settlement	1534	Sinkhole	307	0	77.1	100
		Settlement	1183	1534		
		Mud Pumping	44	0		

**Figure 11:**
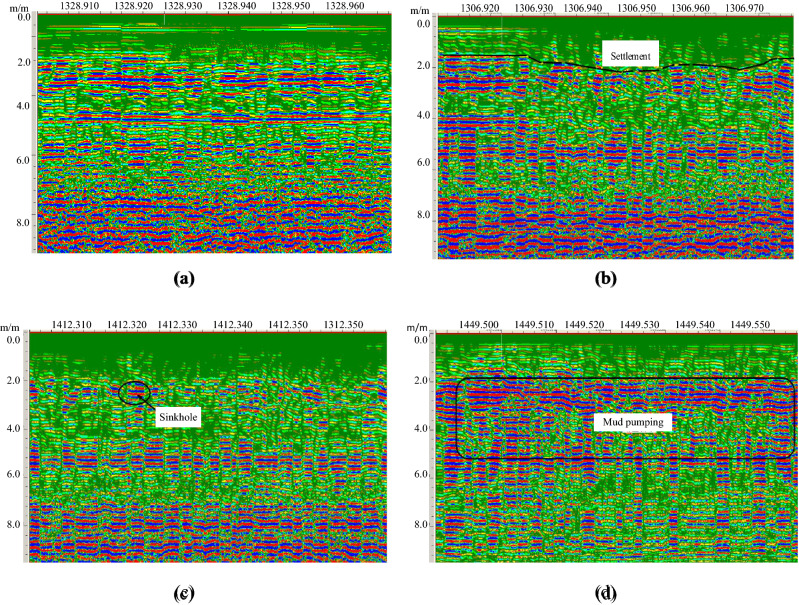
GPR images of subgrade defects. (**a**) normal subgrade, (**b**) settlement, (**c) **sinkhole, (**d**) mud pumping.

To verify the method to identify the subgrade defects, Daqin railway is detected by GPR, as shown in Fig. 11. The normal subgrade is shown in Fig. 11a, and there are some defects, such as settlement (Fig. 11b), sinkhole (Fig. 11c) and mud pumping (Fig. 11d), in the railway sungrades. Figure 11b shows an obvious semi-parabolic on the edge of the stage and line feature at the bottom. Figure 11c shows the phase axis is lower than normal axis, and the range is relatively small. The defects are inferred to be the remains of the artificial mining cave. Under long-term traffic loading and water erosion conditions, the structure of rock and soil is gradually destroyed, and its load-carrying capacity gradually decreases, eventually leading to collapse. The collapse forms a loose area, which results in subgrade settlement, and water-enriched regions form mud pumping. Figure 11d is strongly reflected signal region, and the axis is not exit.

## Conclusion

The railway subgrade defects present sparse in radar images, which meets the requirements of sparse theory. The demixing points, energy, and variance per block are obtained as time domain eigenvalues, and the energy spectrum of the wavelet multiscale spatial are acquired and made up of data dictionary. The optimal sparse radar feature is established based on L1 minimum norm method.

Fuzzy C-means (FCM) and generalized regression neural network (GRNN) are used as the recognition algorithms for subgrade defects. FCM-GRNN simulation and field experiments show that the classification accuracy of sinkhole, mud pumping, and settlement is 100, 100, 59.1%, respectively.

This study combines sparse theory with field experiments and obtains sparse features to identify the subgrade defects. The identification method overcomes the influence of redundant data and promotes GPR application for the detection of railway subgrade defects. However, the classification accuracy of settlement is relatively low. Hence, the identification methods for settlement should be further discussed.

## Data Availability

All data, models, and code generated or used during the study appear in the submitted article.

## Abbreviations

GPR: Ground penetrating radar
FCM: Fuzzy C-means
GRNN: Generalized regression neural network
STFT: Short-time Fourier transform
CS: Compressed sensing

## Code availability

Source code implementing the algorithm described in this work can be obtained from the following git repository: http://www.ilovematlab.com/. This software is written in Matlab (version R2016a). It should run on any modern Linux x86 computer supporting the aforementioned packages.
